# Telemedicine and anaesthesia

**DOI:** 10.4103/0019-5049.65357

**Published:** 2010

**Authors:** Veena Chatrath, Joginder Pal Attri, Raman Chatrath

**Affiliations:** Associate Professor, Department of Anaesthesia, Govt Medical College, Amritsar, Punjab, India; 1 Assistant Professor, Department of Anaesthesia, Govt Medical College, Amritsar, Punjab, India; 2 Senior Consultant Anaesthesiologist, Department of Anaesthesia and Critical Care, KD Hospital, Circular Road, Amritsar, Punjab, India

**Keywords:** Telecommunication, telemedicine, video-conferencing

## Abstract

Telemedicine is the use of electronic information and communication technology to provide and support healthcare when distance separates the participants. India is characterised by low penetration of healthcare services where primary healthcare facilities for rural population are highly inadequate. The majority of doctors practice in urban and semi-urban areas, whereas the major proportion of population lives in rural areas. This calls for the innovative methods for utilisation of science and technology for the benefit of our society. There are few reports in the literature which support the use of telemedicine technology for pre-operative assessment, intra-operative consultation, monitoring and post-operative follow-up, which is discussed in this article.

## INTRODUCTION

Telemedicine has been defined as the use of telecommunications to provide medical information and services. It may be as simple as two health professionals discussing a case over the telephone or as sophisticated as using satellite technology to broadcast a consultation between providers at facilities in two countries using video-conferencing equipment or robotic technology. WHO defines telemedicine as ‘The delivery of healthcare services, where distance is a critical factor, by all healthcare professionals using information and communication technologies for exchange of valid information for diagnosis, treatment and prevention of disease and injuries, research and evaluation and for the continuing education of healthcare providers, all in the interests of advancing the health of individuals and their communities’.[1,2]

Telemedicine generally refers to the use of intercommunication technology (ICT) for the delivery of clinical care.

## HISTORY OF TELEMEDICINE

While the explosion of interest in telemedicine over the past 4 or 5 years makes it appear that the use of telecommunication technology is relatively new, the truth is that telemedicine has been in use in some form or other for over 30 years. Nebraska Psychiatric Institute was one of the first facilities in the country to have a closed-circuit television in 1955. In 1967, Massachusetts General Hospital linked up with Logan Airport to provide occupational health services to airport employees and to deliver emergency care and medical attention to travellers by using two-way audio visual microwave circuits.[2,3] In 1971, Alaska Biomedical Demonstration Project linked 26 sites using NASA satellite technology.[4]

### Types of telemedicine

Telemedicine is practised on the basis of two concepts:

1. Real time (synchronous)
2. Store and forward (asynchronous).

#### Real time (synchronous) telemedicine

Real time telemedicine could be as simple as a telephone call or as complex as robotic surgery. It requires the presence of both parties at the same time and a communication link between them that allows a real time interaction to take place. Video-conferencing equipment is one of the most common forms of technology used in synchronous telemedicine. There are also peripheral devices which can be attached to a computer or the video-conferencing equipment which can aid in an interactive examination, e.g. a tele-otoscope allows a remote physician to see inside a patient’s ear, a tele-stethoscope allows a consulting remote physician to hear the patient’s heart beats.[5,6]

#### Store and forward telemedicine (asynchronous)

Store and forward telemedicine involves acquiring medical data like medical images, biosignals, etc. and then transmitting these data to a doctor or medical specialist at a convenient time for assessment offline. It does not require the presence of both parties at the same time. Dermatology, radiology and pathology are common specialties that are conducive to asynchronous telemedicine. A properly structured medical record preferably in electronic form should be a component of this transfer. Tele-radiology, the sending of x-rays, CT scans or MRIs (store and forward images), is the most common application of telemedicine in use today.[6]

Telemedicine is most beneficial for populations living in isolated communities and remote regions and is currently being applied in virtually all medical domains. Images of pathology slides and digital images of skin conditions may be sent from one location to another for diagnostic consultation.

Video-conferencing equipment at both locations allows for a real time consultation to take place. The technology has decreased in price and complexity over the past 5 years and many programs now use desktop video-conferencing systems. There are many configurations of an interactive consultation, but most typically it is from an urban to rural location. It means that the patient does not have to travel to an urban area to see a specialist, and in many cases the technology provides access to specialty care when none has been available previously.[7,8]

The staff operating the video-conferencing equipments should have a fundamental understanding of the concepts and principles which underlie the telemedicine technology. For this they have to do a certification course. There is a 1-month certified course from CDAC (Centre for Development of Advanced Computing), Mohali, Punjab. They can also upgrade their knowledge with a 1-day or 12-h CME (Continuing Medical Education). The ICT University’s (Information and Communication Technologies University) online telemedicine course is designed to help healthcare providers incorporate telemedicine techniques into daily clinical practice.

## ADVANTAGES OF TELEMEDICINE

Providing healthcare services via telemedicine offers many advantages. It can make specialty care more accessible to underserved rural and urban population through the following means:

- Video consultation from a rural clinic to a specialist can alleviate prohibitive travel and associated cost for patients. It increases access to specialty consultations and/or second opinion for better diagnosis, treatment and prognosis. One-to-one dialogue with a superspecialist expert and immediate guidance for the course of treatment in the case of emergency can be taken up.
- Post-operative follow-up and rehabilitation support can be provided through telemedicine.
- Remote ICCU-CCU can be monitored.
- Video-conferencing also opens up new possibilities for continuing education or training for isolated or rural health practitioners who may not be able to leave a rural practice to take part in professional meetings or educational opportunities.
- The use of telemedicine can also cut the cost of medical care for those in rural areas.[8,9]

## BARRIERS TO TELEMEDICINE

Many potential telemedicine projects have been hampered by the lack of appropriate telecommunication technologies. Regular telephone lines do not supply adequate bandwidth for most telemedicine applications. Many rural areas still do not have cable wiring or other kind of high-bandwidth telecommunication access required for more sophisticated uses. There is also a lack of funds for developing state-of-the-art telemedicine infrastructure. Some private corporation and telecommunication companies are stepping in to fill the void; however, pressure on the appropriate government and legislative agencies is needed before more funds will become available. Many states will not allow out-of-state physicians to practise unless licensed in their states.[9,10]

## TELEMEDICINE INFRASTRUCTURE

This includes minimum standard for all the hardware and software used in the telemedicine system.

Hardware: This includes the telemedicine platform, clinical devices, video-conferencing unit, and communication hardware.

Software: It consists of an operating system, licensed telemedicine software with an appropriate user interface (UI), a back end database with the mandatory tables/fields if applicable. Many software packages are used of which Sanjivini software is most commonly used in India.

**Figure 1:**
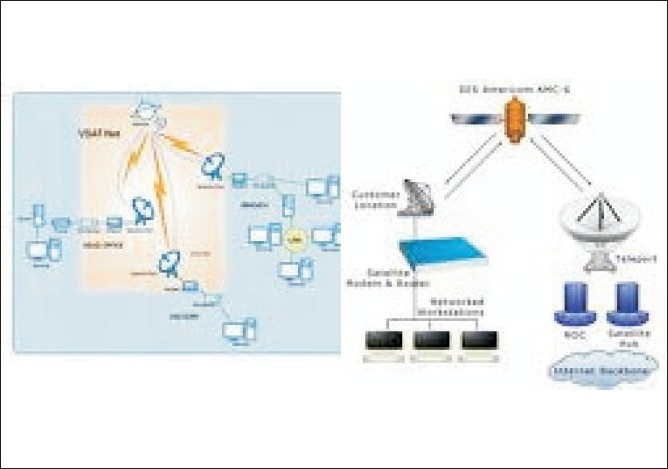
VSAT (Router, Dish antenna, Modem, DAMA unit)

**Figure 2:**
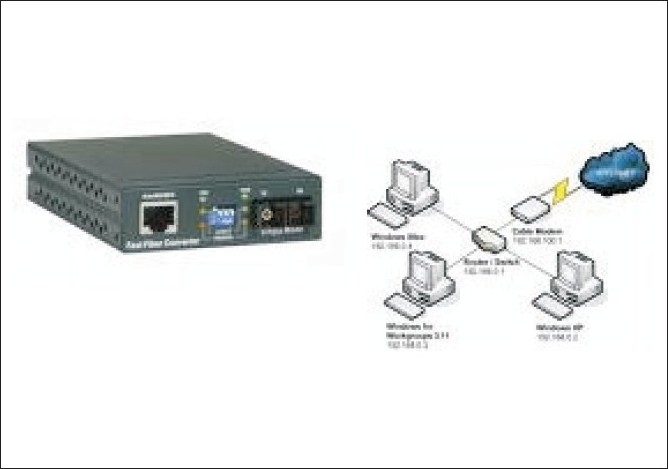
Terrestrial (Media Converter, Router, STM, Power back up for one hour, 6 U Rack)

**Figure 3:**
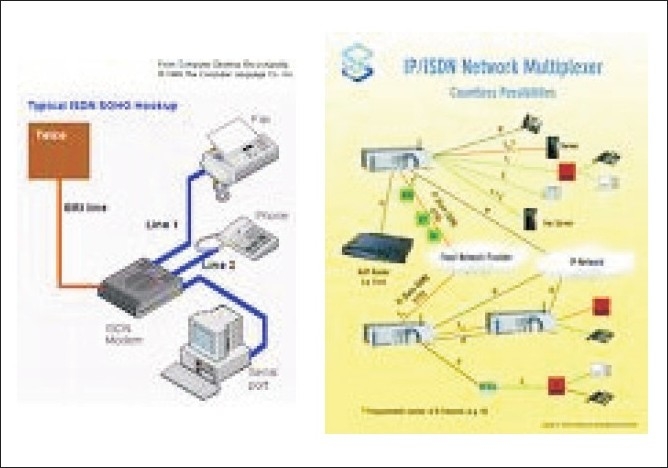
ISDN (ISDN modem, Data/ fax/Voice modem, USB Hub, 10/100 Switch, ISDN phone)

Connectivity: Options for telemedicine services are [Figures 1–3]

- VSAT (very small aperture telephone network)
- PSTN (public services telephone network)
- ISDN (integrated services digital network)
- Leased line
- Wireless LAN/WAN (wireless local area network/wide area network).

### Video-conferencing

**Figure 4:**
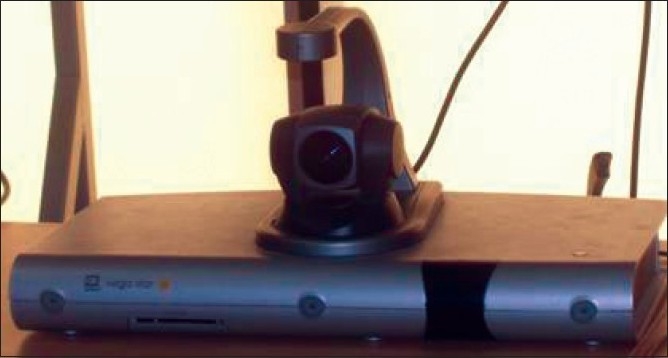
VC operating protocol

**Figure 5:**
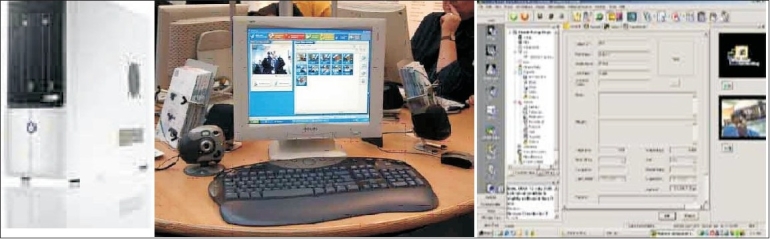
Telemedicine equipment

Video-conferencing is becoming quite popular among hospitals. By using this technology, doctors can help patients who are unable to come to a hospital. Telemedicine has turned out to be a blessing for patients who were otherwise deprived of the best treatment. Broadband has come as a big boost to telemedicine. With the availability of broadband, India is now becoming a hot destination for telemedicine as it is a win-win situation for both the patients and hospitals. The primary objective of telemedicine is to provide quality healthcare assistance to every patient. Video-conferencing also ensures that the patients are treated irrespective of their financial condition and this makes it popular in most countries. It is expected that home-based patient monitoring is also going to become popular, as will remote surgery, which will involve surgery via robotics, with the surgeon in one location and the patient in another[10] [Figures 4 and 5].

## APPLICATION OF TELEMEDICINE IN ANAESTHESIA

Telemedicine enables the delivery of healthcare irrespective of a geographic location or ability to travel to tertiary healthcare centres. In India, a significant population lives in remote regions away from tertiary care centres. Telemedicine can potentially reduce travel costs and improve accessibility to healthcare. Although telemedicine has been used by other medical and surgical specialties, there have been a very few reports in the literature evaluating telemedicine technology for anaesthesia consultation.

### Pre-admission anaesthesia consultation using telemedicine technology

Preadmission anaesthesia consultations using telemedicine technology can be successfully performed. Informed consent in some form is considered as a requirement for telemedicine and is regarded as a separate process from consent for treatment. Written informed consent is taken from the patient by the healthcare provider in which the patient is informed how the video-conferencing technology will be used to affect such a consultation. The patient is explained the fact that his/her healthcare information may be shared with other individuals for scheduling and billing purposes. The risk and benefits of the teleconferencing consultation including interruptions, unauthorized access and technical difficulties are well discussed with the patient. The patients are also explained that they will be finally reviewed by the anaesthesiologist on site, before proceeding for telemedicine consultation.[11,12]

A pilot study was done in Canada, where a significant population lives in remote regions away from tertiary healthcare centres. In this study the technical aspects and implementation of telemedicine consultations are reported.[12]

## TELEMEDICINE TECHNOLOGY

The University Health Network in Toronto developed a partnership with Northern Ontario Remote Telecommunication Health Network to provide telemedicine clinical consultations to residents of central and northern Ontario in Canada.

### Telemedicine set-up

Both the sites are equipped with video-conference television monitors and cameras that allow live two-way communication.

### The remote (patient) site

A light source is connected to two analogue cameras. The first camera functions as the room camera and the second serves as the airway camera for intraoral views. A digital electronic stethoscope permits the transmission of heart and lung sounds.

### Connection

The north network uses the existing communications network smart systems for health provided by Bell Canada operating with a bandwidth of 384 Kbps.

### The consultant site

The set-up incorporates a monitor, a camera, a desktop computer and a digital stethoscope. When connected to the remote site, the anaesthesiologist can visualize, hear and auscultate the patient using the digital stethoscope system. The anaesthesiologist inserts the digital stethoscope, ear pieces in exactly the same manner as a conventional stethoscope. The audible frequency range can be varied manually depending upon whether high pitched or low pitched sounds are being auscultated. An anaesthesiologist is present at the consultant site while an attending doctor/nurse can accompany the patient at the remote site during anaesthesia consultation. The anaesthesiologist takes a history from the patient as in a conventional consultation. Examination of the airway, respiratory and cardiovascular system is performed. Mouth opening and the Mallampati score is assessed using the airway camera. The patient is then turned and a side-view visual assessment of the airway profile, thyromental distance and neck movement is made using the room camera. The nurse at the remote centre is instructed on the positioning of the stethoscope on the patient’s chest and precordium. The rest of the consultation is conducted as per the conventional consultation.[12,13]

The relevant investigations ordered by the consultant anaesthesiologist during the telemedicine consultation are noted by the attending doctor and are reviewed by the anaesthesiologist on the day of surgery.

General physical examination, including the airway assessment and site for venous access are also checked by the anaesthesiologist on the day of surgery, because the initial findings noted on telemedicine consultations may vary. So, a final review regarding history, physical examination and investigations is important before giving anaesthesia.

There are several limitations to the telemedicine consultation process. First, there is privacy concern for the patients because they are being asked to provide personal details and exposure of chest is asked for consultation. Second, telemedicine consultation does not permit any physical contact between the physician and the patient. Third, the patient and the anaesthesiologist cannot speak at the same time. In store and forward telemedicine, medical data of the patient like history, investigations and medical images are acquired on the electronic medical record and then transmitted to the consulting anaesthesiologist at a convenient time for assessment offline. So in this technology there is no verbal contact between the patient and the consultant anaesthesiologist. The important findings on general physical examination and airway assessment may be missed with this technology.[13–15]

## PAEDIATRIC LIVER TRANSPLANT USING TELEMEDICINE TECHNOLOGY

Till date, the application of telemedicine to anaesthesia (tele-anaesthesia) has been limited. The use of telemedicine to support two cases of elective living-related paediatric liver transplant performed at the Narayana Hrudayala Institute of Medical Sciences in Bangalore (India) with pre- and intra-operative consultation provided by physicians at the children’s hospital of Philadelphia is reported.

They directed two liver transplants in patients aged 4 years and 16 months. The equipment used in Bangalore consisted of a video camera, a video conference device by Polycom and an ISDN (Integrated Services Digital Network) line with a 128 Kbps bandwidth. ISDN allows voice and data to be transmitted simultaneously using end-to-end digital connectivity.

A week prior to the date of surgery, GoToMeeting online software was used to present a lecture on paediatric liver transplantation to the anaesthesia providers in Bangalore by anaesthesiologists of Philadelphia. GoToMeeting also served as a back-up communication system during the anaesthetic course. This is the first application of teleconferencing and a web-based connection to facilitate remote anaesthesia management in real time. There are however several challenges such as time zone difference, medical liability, setting adequate backup system, obtaining informed consent and maintaining an ongoing patient’s record at both locations simultaneously. Despite these issues tele-anaesthesia remains an option to provide intra-operative consultation or medical direction of an anaesthetic expert at a distant location.[16,17]

## TELE THERAPEUTIC DRUG ADMINISTRATION

A pilot study was done to investigate the feasibility of an EEG-controlled closed loop administration of propofol over a long distance of about 200 km. A teletherapeutic propofol infusion was performed during TIVA with propofol in 11 patients undergoing general surgery. The teletherapeutic system consisted of a computer at the patient site in Munich and a computer at the control site in Erlangen which were connected via the internet through a virtual private network. The patient’s EEG was sent to the control site computer where the median frequency (MEF) of the EEG power spectrum was calculated and the dose of propofol was adjusted according to the MEF.[18]

## OFFSITE (REMOTE ) CRITICAL CARE PATIENT MONITORING

The doctor in the local ICU connects the patient to the ICU or CCU of specialist centre where the expert cardiologist and critical care specialists review the patient’s condition online and provide expert opinion to local ICU or CCU doctor in managing the patient with the best of the clinical practice.[19–21]

## HOME MONITORING

Patients with chronic ailments can have their follow-up consultations with their respective consultants while sitting in their homes or work places. The patient is given a device that needs to be connected to an ordinary telephone line and during the consultation this device transmits the ECG and other relevant clinical parameters for analysis.[22]

## CONCLUSIONS

Telemedicine is becoming more readily accessible and affordable and its various applications are growing rapidly with promising results. It has proved to be very practical and effective in providing patient-centred specialty care to remote locations. Telemedicine has been reliable for pre-operative assessment, pre-operative teaching, intra-operative consultation and post-operative follow-up for common anaesthesia and surgical problems. Tele-anaesthesia, although still in its infancy, may be a positive development in providing anaesthesia/surgical care to rural and remote locations. The technology of a telemedicine application may be very simple, but a real need for communication/collaboration can make it a full success. As technology continues to improve and we all become better educated consumers and professionals, the potential of telemedicine and its role in healthcare will be tremendous.
